# Lymphoid cell development from fetal hematopoietic progenitors and human pluripotent stem cells

**DOI:** 10.1111/imr.13197

**Published:** 2023-03-20

**Authors:** Shicheng Sun, Kevin Wijanarko, Oniko Liani, Kathleen Strumila, Elizabeth S. Ng, Andrew G. Elefanty, Edouard G. Stanley

**Affiliations:** ^1^ Murdoch Children's Research Institute The Royal Children's Hospital Parkville Victoria Australia; ^2^ Department of Paediatrics, Faculty of Medicine, Dentistry and Health Sciences University of Melbourne Parkville Victoria Australia; ^3^ The Novo Nordisk Foundation Center for Stem Cell Medicine (reNEW), Murdoch Children's Research Institute Parkville Victoria Australia

**Keywords:** fetal lymphopoiesis, immune ontogeny, PSC differentiation, RAG genes

## Abstract

Lymphoid cells encompass the adaptive immune system, including T and B cells and Natural killer T cells (NKT), and innate immune cells (ILCs), including Natural Killer (NK) cells. During adult life, these lineages are thought to derive from the differentiation of long‐term hematopoietic stem cells (HSCs) residing in the bone marrow. However, during embryogenesis and fetal development, the ontogeny of lymphoid cells is both complex and multifaceted, with a large body of evidence suggesting that lymphoid lineages arise from progenitor cell populations antedating the emergence of HSCs. Recently, the application of single cell RNA‐sequencing technologies and pluripotent stem cell‐based developmental models has provided new insights into lymphoid ontogeny during embryogenesis. Indeed, PSC differentiation platforms have enabled de novo generation of lymphoid immune cells independently of HSCs, supporting conclusions drawn from the study of hematopoiesis in vivo. Here, we examine lymphoid development from non‐HSC progenitor cells and technological advances in the differentiation of human lymphoid cells from pluripotent stem cells for clinical translation.

## 1 INTRODUCTION

The lymphoid hematopoietic lineage gives rise to a broad spectrum of immune cell types, including T cells, B cells, and natural killer (NK) cells as a subtype of innate lymphoid cells (ILCs). These cell types are thought to arise from long‐term hematopoietic stem cells (HSCs) that reside in the adult bone marrow (BM), later maturing in primary lymphoid organs, namely the thymus and the BM. Following lymphoid commitment, hematopoietic progenitors differentiate to functional T and B cells through a process that incorporates DNA recombination events that generate antigen‐specific receptors. In this context, a hallmark of adaptive immune cell development is the expression of the RAG genes (RAG1 and RAG2), encoding proteins that mediate rearrangement of the T cell and B cell receptor genes. While T cell receptors (TCRs) are always associated with the cell surface, BCRs, composed of heavy and light polypeptide chains, can exist as both membrane‐bound BCRs and secreted antibodies. The generation of lymphocyte antigen receptors is highly dependent on successful gene rearrangements of TCR and Ig genes by V(D)J recombinase which are composed of RAG1 and RAG2. As such, expression of RAG genes is a prerequisite for the generation of adaptive immune cells, making RAG expression a key milestone in the development of adaptive immune cells.

T and B cell receptors enable the development of adaptive immunity, which accelerates after birth as adaptive immune cells are exposed to environmental antigens and pathogens. An alternative pathway of lymphoid development without DNA recombination generates innate lymphoid cells that are now known to have critical roles in coordinating the adaptive immune response by secreting cytokines and eliminating pathogens and malignant cells.

However, transplantation of adult HSCs does not reconstitute all lymphoid cell types,^1^, ^2^ supporting the idea of an HSC‐independent lymphoid ontogeny during embryonic development. Indeed, in early human embryos, lymphoid cells can be detected before the formation of functional primary lymph organs and HSCs.^3^, ^4^, ^5^ Furthermore, experiments in the mouse suggest that lymphoid cells can be generated from non‐HSC progenitors^6^, ^7^, ^8^ including the erythroid/lympho‐myeloid progenitor (EMPs or LMPs) and pre‐HSCs, found in the mouse yolk sac (YS) and aorta‐gonad‐mesonephros (AGM), respectively. Knowledge of early embryonic human lymphopoiesis is critical for understanding pathological conditions, such as childhood leukemia and autoimmune disorders, and for developing immunotherapies.

The study of lymphoid cell development during human embryogenesis has proved challenging due to the scarcity of fetal material available for analysis. Nevertheless, recent single cell‐RNA sequencing (scRNA‐seq) of human embryo tissue has provided important insights into the potential differentiation trajectories taken by lymphoid cells before and after the emergence of HSCs. Research using scRNA‐seq technology has generated cell transcriptomic datasets for human fetal hematopoiesis and lymphopoiesis that incorporates rare human fetal tissues, such as the yolk sac,^3^, ^4^ the AGM,^4^ fetal liver,^4^, ^5^ and the thymus.^9^, ^10^ Complementing scRNA‐seq findings, in vitro analyses can be performed by differentiating human PSCs and examining the lymphoid potential of different non‐HSC progenitors that lack long‐term repopulating capacity. In addition, the ability to genetically modify PSCs has enabled the generation of gene‐specific reporter lines to assist in monitoring the emergence of key lymphoid populations during differentiation.^11^, ^12^ This knowledge has contributed to the development of strategies for producing functional human lymphoid immune cells that hold great promise for clinical translation.

Here, we present an overview of lymphoid development from non‐HSC populations in vivo and in vitro, with a focus on the human system. We have closely examined T, B, and NK cell development but have not delved too deeply into the ontogeny of non‐NK ILCs, NK‐T cells nor MAIT cells, which have been recently reviewed elsewhere.^13^, ^14^ We also discuss differentiation methods for the generation different types of lymphoid cells from hPSCs and their clinical implications in disease modeling and cell therapies.

## 2 LYMPHOID POTENTIAL IN DISTINCT EMBRYONIC HEMATOPOIETIC WAVES

**FIGURE 1:**
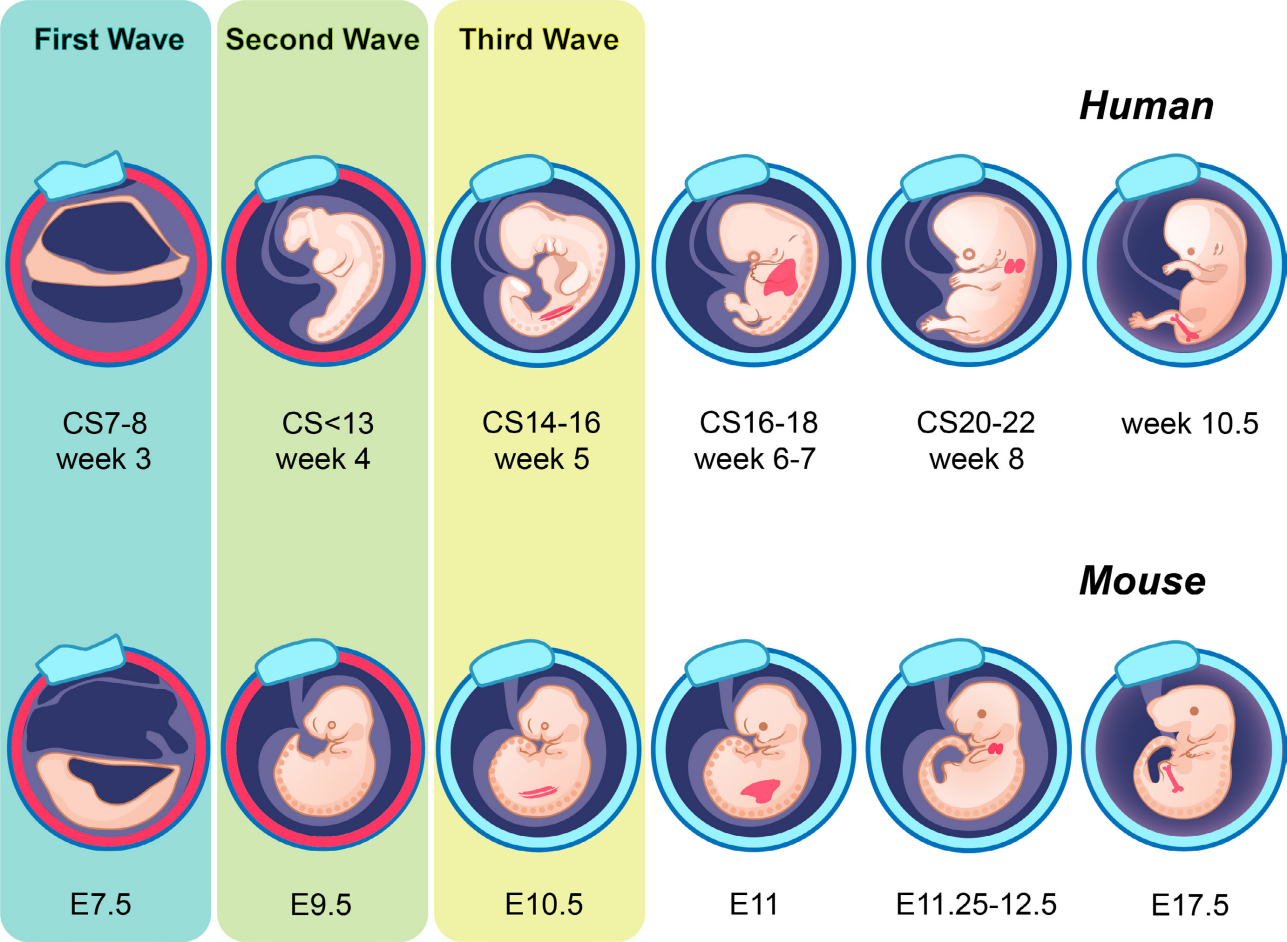
Multiple waves of embryonic hematopoiesis. Hematopoietic waves of human (upper series, in Carnegie Stages, CS) and mouse (lower series, in embryonic day, E). Key embryonic hematopoietic events in each tissue are marked in red. These include hematopoiesis in the YS (CS7‐12 or E7.5‐9.5), AGM (CS14/16 or E10.5), hematopoietic stem cell colonization of the fetal liver (CS16‐18 or E11), thymus (CS20‐22 or E11.25), and bone marrow (embryonic week 10.5 or E17.5). The correspondence between Carnegie stages (CS) and weeks is approximate.

In both mouse and human, embryonic hematopoiesis occurs through multiple, temporally overlapping, independent waves, each of which creates hematopoietic cells with distinct developmental potency. For both species, embryonic hematopoiesis can be classified into three major waves, defined by their anatomical location and their capability of forming specific lineages (Figure 1). In the context of this review, each of these waves displays a unique potential to generate lymphoid cells.

**FIGURE 2:**
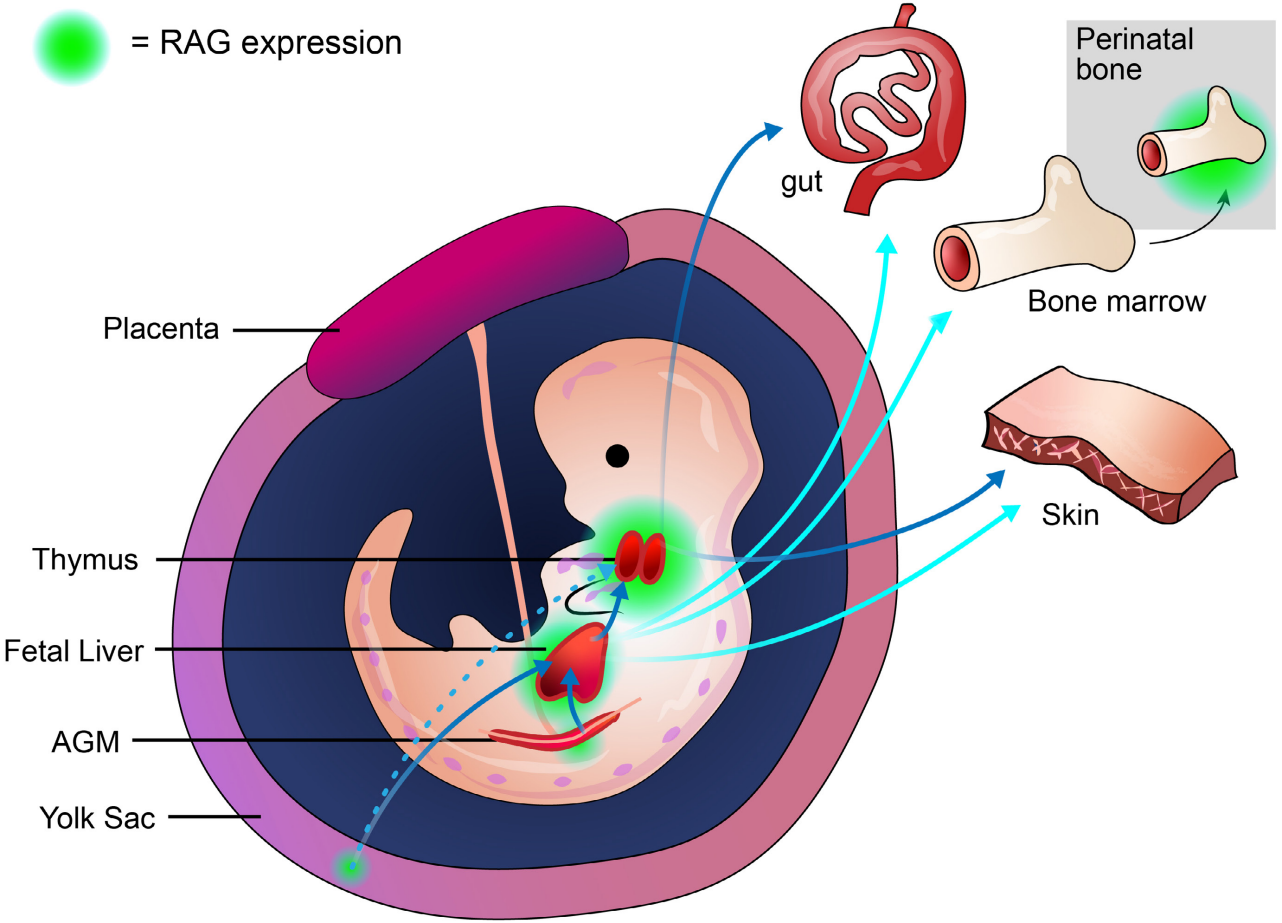
Emergence and trafficking of hematopoietic and lymphoid progenitor cells during embryogenesis. Green shading indicates intensity of RAG activity in specific hematopoietic and lymphoid tissues. Rare and weak RAG activation is found in mouse yolk sac tissues, which is followed by the detection of RAG^+^ cells in the hematopoietic tissues of the AGM and the fetal liver, and the primary lymphoid organs of the thymus and the bone marrow. Arrows indicate hypothesized migration of hematopoietic/lymphoid progenitor cells to different locations to generate functional lymphoid cells, including unconventional T cells formed in the skin and gut during early embryonic development.

In mice, the first wave of hematopoiesis, also known as primitive hematopoiesis, is detected in the yolk sac at embryonic day 7.5 (E7.5) and mainly produces primitive erythrocytes that support the growing embryo.^15^ Definitive hematopoiesis begins independently in the yolk sac at E9.5 and in the AGM at E10.5 as the second and the third waves, respectively.^16^, ^17^ Yolk sac definitive hematopoiesis generates erythroid‐myeloid progenitors (EMPs), and in vitro colony forming assays show yolk sac EMPs can produce erythroid, granulocyte, and macrophage lineages. Similarly, explant cultures incorporating stromal cells indicate that yolk sac progenitors, until E9‐9.5, have the potential to generate T and B lineage cells in vitro.^6^, ^7^ However, it remains unclear whether this lymphoid potential is realized in vivo, as the primary lymphoid organs, which would support ongoing lymphoid development, are not fully formed at this timepoint. Indeed, RAG1, a signature gene of adaptive immune cell development, is rarely and lowly expressed in the E10 YS, which is followed by a robust increase in the embryo proper^18^ (Figure 2). Initial thymic seeding cells observed between E11.25‐11.5 are RAG1^+^. These cells have a similar gene expression profile to lymphoid‐myeloid progenitors (LMPs) found in the E10.5‐11.5 FL,^19^ suggesting this tissue represents an intermediary in the genesis of early thymic lymphoid progenitors. Nevertheless, it is possible that thymic seeding cells could originate from the YS and seed the thymus, with or without migrating through the FL. Last, there is evidence from in vitro studies using PSCs (discussed below) showing that non‐HSC progenitors with an intraembryonic like phenotype can also generate lymphoid progenitors.

The AGM is a well‐established site known to produce long‐term transplantable HSCs. Before colonizing the bone marrow (BM), HSCs are believed to transition through a pre‐HSC stage in the AGM and the fetal liver. In mice, recent fate mapping analysis shows that pre‐HSCs at E10.5 within AGM intra‐arterial hematopoietic clusters develop contemporaneously with embryonic multipotent progenitors, called eMPPs, that hold lymphoid potential with lifelong contribution in adults.^8^ Thus, although pre‐HSCs and eMPPs emerged within a similar time window during fetal hematopoiesis, eMMPs do not repopulate the hematopoietic system after transplantation. These experiments performed with mice established a hierarchy of lymphoid potential of different progenitor cell types emerging from multiple hematopoietic waves (Figure 2).

In humans, recent scRNA‐seq analysis of an entire gastrulating human embryo identified two independent hematopoietic populations at Carnegie stage (CS) CS7 (corresponding to mid‐late gastrulation, approximately 2.5 weeks post fertilization), showing characteristics of YS primitive erythroid progenitors and EMPs, without a noticeable lymphoid population.^20^ An independent analysis of this dataset showed the presence of a mesoderm population that co‐expressed the Vascular Endothelial Growth Factor Receptor (KDR), a haemato‐endothelial marker, and Glycophorin A/B (CD235a/b), a surface marker identified on early hemogenic progenitors and erythroid cells. Experiments using human PSC differentiation showed that KDR^+^CD235a/b^+^ mesodermal cells are able to give rise to erythroid, myeloid, NK and γδT cell lineages in vitro,^21^ consistent with the analysis of YS hematopoiesis in mice discussed above.^7^, ^22^ However, an experiment comparing lymphoid potential of hematopoietic progenitors isolated from bona fide human YS and intraembryonic aortic tissues (CS 8‐16) (approximately 3‐6 weeks) showed that YS progenitors did not generate T cells or B cells, but were able to generate NK cells. It was progenitors from the embryo proper that produced CD19^+^ B, CD4^+^ T and CD56^+^ NK cells.^23^ These discordant observations concerning T cell differentiation could reflect the ability of PSC differentiation systems to generate a non‐physiological MPP cell type that can give rise to T cells, or the exposure of essentially physiological cell types to environmental conditions that would not normally be encountered during embryogenesis in vivo, such as the NOTCH ligand‐expressing animal stromal cells.^24^, ^25^ Nevertheless, human PSC‐based models suggest that RAG1^+^ lymphoid progenitors can emerge directly from an AGM‐like definitive hemogenic vasculature,^12^ potentially reflecting a lymphoid developmental pathway of physiological relevance^26^ (Figure 2). On the contrary, no studies have found B cell potential associated with human YS or YS‐like hematopoiesis, which is considered to be restricted to AGM‐derived definitive hematopoiesis.

Recent reports suggest the earliest human cells with lymphoid potential are present in the YS of 4‐week‐old embryos.^3^ Thus, although few YS progenitors express the IL7 receptor (IL7R), reinforcing the argument that cells from this site have limited lymphoid potential, ILC progenitors can be detected at week 4 (CS12), followed by detection of differentiated ILCs and NK cells at week 5 (CS14).^3^ Indeed, yolk sac‐derived SPINK2^+^IL7R^+^ cells, representing lymphoid‐myeloid progenitors, populate the FL at CS14 before the appearance of HSCs.^3^, ^4^ These IL7R^+^ cells were identified from a subset that expressed CD34 and SPINK2 but no other HSPC associate genes (HLF, HOXA9, and HOXB9). In this context, the absence of HOXA9 expression is potentially indicative of the extra‐embryonic origins of these cells. Multilineage hematopoiesis is observed in the FL at CS17 (approximately week 6). At weeks 7‐8 (CS19‐21), FL lymphoid cells express genes including GATA3 (ILC/T), KLRB1 (ILC/NK), CD3D (T), CD7 (lymphoid), and JCHAIN (B).^4^ At this stage of development, ILCs, γδ T cells, and DN thymocytes are detectable in the thymus primordium that is not yet considered functional for thymopoiesis.^10^

**FIGURE 3:**
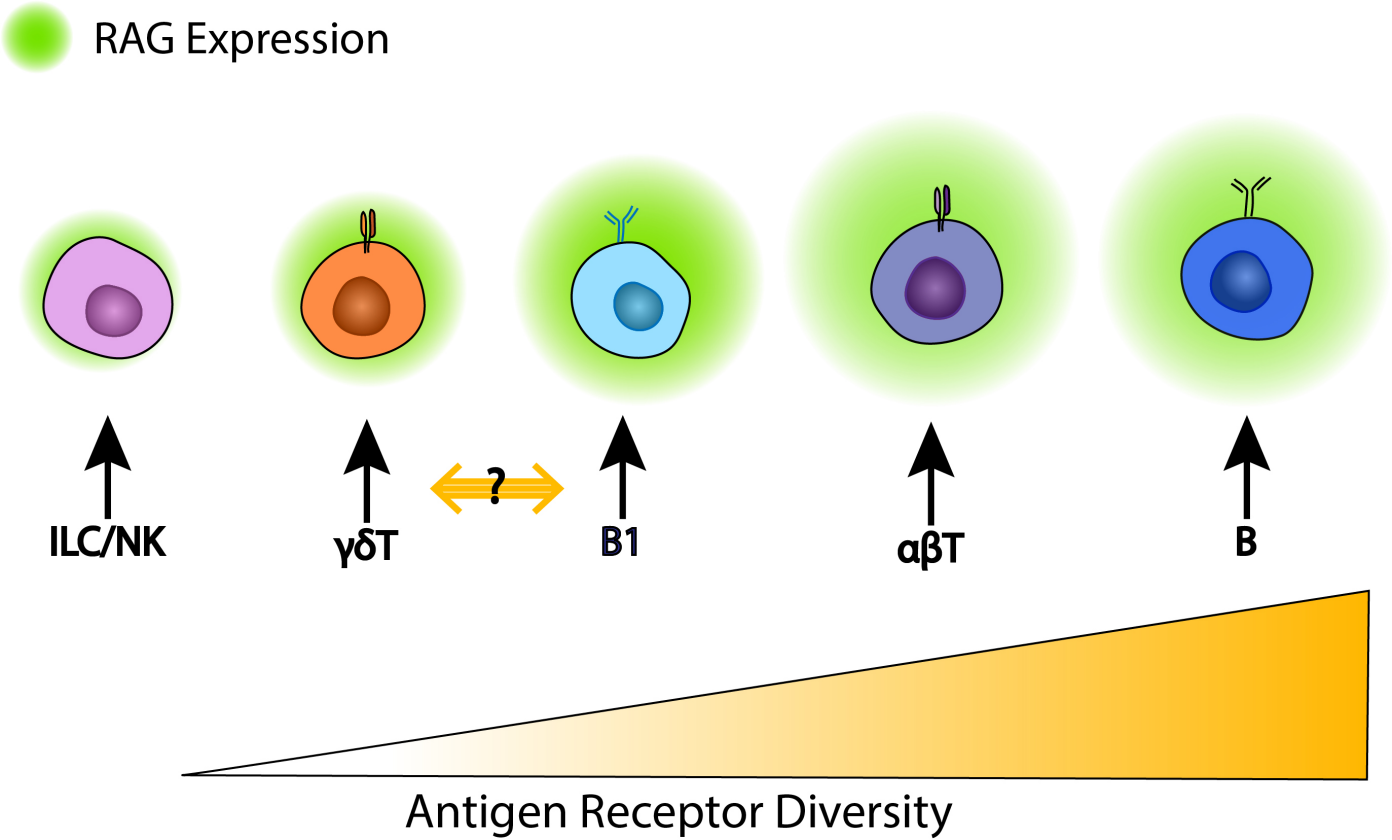
Variation of RAG expression intensity, indicated by green shading, in relation to the diversity of antigen‐specific receptor expression in different lymphoid cell types. The relative diversity of antigen specificity receptors of γδ T cells vs B1 B cells has not been unequivocally determined (indicated by “?”).

These observations highlight difficulties in determining the exact origins of early lymphoid populations observed in the fetal liver shortly after the onset of hematopoiesis in the AGM, particularly for cells of the T/innate lymphoid lineages, which could potentially originate from either the yolk sac or a non‐HSC AGM‐derived precursor. Nevertheless, TCR bearing lymphoid cells appearing earlier in development have less receptor diversity than those appearing later (Figure 3), paralleling observations from mouse studies. In turn, the reduced TCR diversity associated with early adaptive lymphoid cells is partly reflected in the less robust and temporally restricted expression of RAG recombinases in their progenitors. Using mouse studies as reference frame, RAG‐mediated recombination and TCR diversity of lymphoid cell types in early human embryos is discussed below.

## 3 LYMPHOID CELLS FROM NON‐HSC PROGENITORS

### 3.1 T cells

**FIGURE 4:**
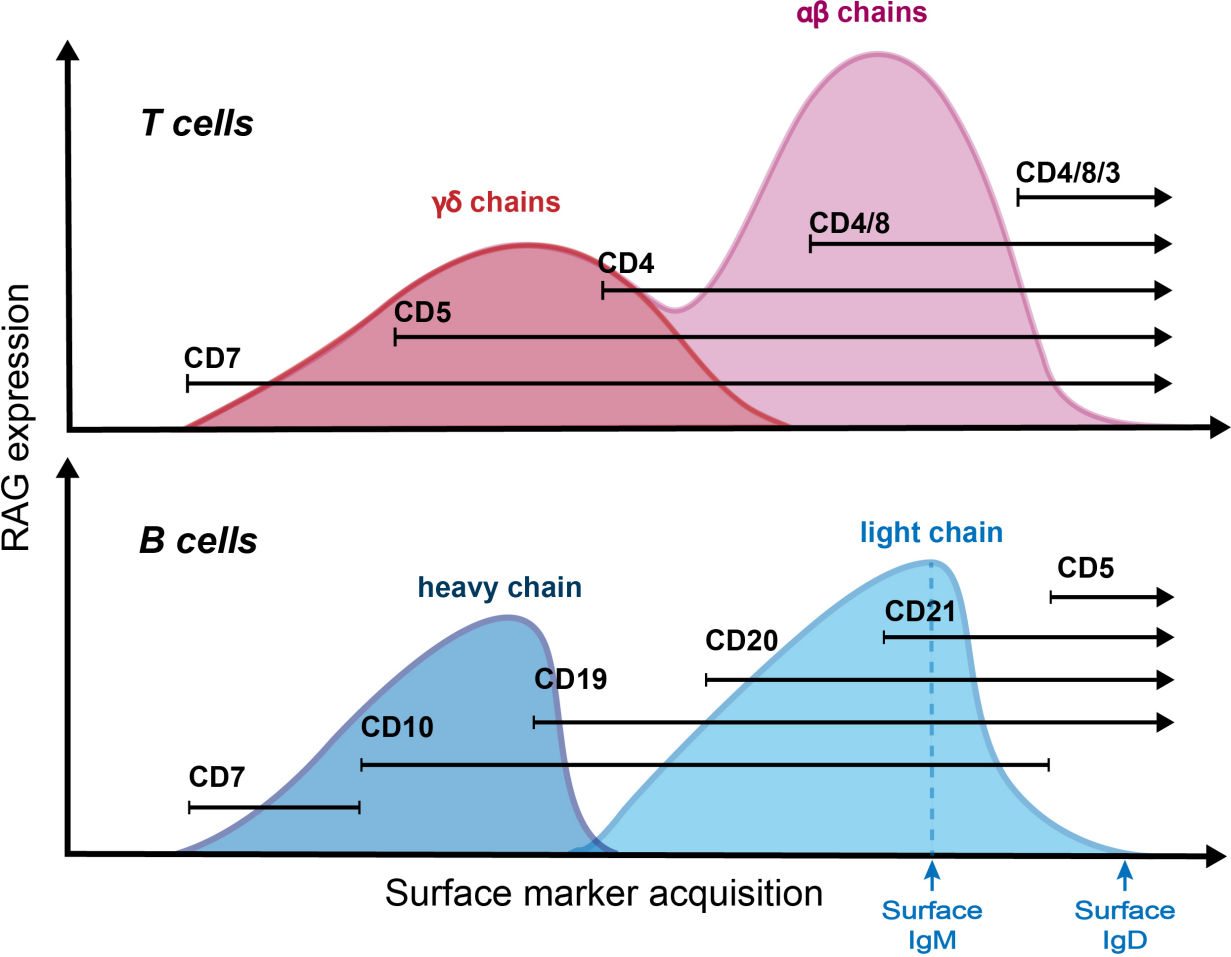
Kinetics and dynamics of RAG expression during T cell (upper) and B cell (lower) development. The schematics show the relation between the level of RAG gene expression in the context of key surface markers used to stage T and B cell development. In scheme for T cells, dark and light pink curves indicate the RAG kinetic and dynamics for γδ T cells and αβ T cells, respectively.

Studies of the developing mouse have established that multiple waves of lymphopoiesis contribute the complexity of innate and adaptive T lineage cells observed in the adult, a phenomenon elegantly demonstrated through the analysis of thymic seeding populations.^27^ A detailed description of these waves is provided in this Issue of *Immunological Reviews* by Cumano and colleagues. A characteristic of these distinctive waves is the relative diversity of T cell receptor genes. T cells found in early embryos are predominantly invariant γδ T cells whose T cell receptor repertoire is drawn from a highly restricted set of V genes and encompasses cells with a reduced spectrum of TCR specificities when compared to αβ T cells. Reflecting this, γδ and αβ T cell subtypes undergo different RAG‐mediated recombination events and have differing developmental trajectories (Figures 3 and 4). The derivation of specific γδ T cell subsets from non‐HSCs in early mouse and human embryos has been established previously.^28^ The lymphoid potential of cells from the YS and AGM of E9‐9.5 Ncx^−/−^ mouse embryos (that lack blood circulation, preventing progenitor cell circulation to between organs) was studied by transplantation into immune deficient mice or using OP9‐DL1 co‐cultures.^7^ These experiments showed that YS cells transiently repopulated the thymus of immune deficient mice and generated T cells with αβ or Vγ5 γδ T cell receptors when cultured on OP9‐DL1 cells.^7^ Using Cdh5^CreERT2^ mice, which enable cells undergoing a transient upregulation of Cdh5 (VE‐Cadherin) during the endothelial‐to‐hematopoietic transition (EHT) to be irreversibly labelled, Gentek et al^22^ tracked lymphoid cells from progenitors arising from early YS EHT (labeled at E7.5) and AGM EHT (labeled at E10.5). This study concluded that a proportion of murine dendritic epidermal T cells arise from a YS endothelial‐hematopoietic transition but not from the AGM. In addition, using Runx1^CreERT2/wt^ Rosa^yfp/w^ mice exposed to Tamoxifen at E8.5, Gentek et al^22^ also found that the majority of dendritic epidermal T cells had their origins in late YS hematopoiesis. This conclusion was supported by work using Sox13 reporter mice, which identified Sox13^hi^ YS progenitors as an origin for γδ17 T cells that maintain barrier function via rapid secretion of IL17 in response to mucosal damage.^29^, ^30^ Moreover, in vitro explant experiments showed that E13.5 mouse FL lympho‐myeloid progenitors cultured on human fetal thymic organ cultures failed to generate Vγ4 γδ T cells but reliably gave rise to αβ and many other γδ T cell lineages. Instead, E10.5 YS cells were able to generate Vγ4 and Vγ5 γδ T cells, consistent with the findings of Gentek et al.^22^, ^30^ Taken together, these studies provide strong experimental evidence that in the mouse, YS progenitors can give rise to T cells with a preference for early γδ T cell lineages.

In humans, Vγ9δ2 T cells in the FL have been identified as early as weeks 5‐6, a time point that proceeds the migration of the HSCs from the AGM to the FL and antedates the first wave of thymic colonization, arguing for the non‐HSC derived extrathymic origin of these cells.^31^ The earliest CD7^+^ lymphoid progenitor cells are found in the thymus at week 7 via scRNA‐seq, and weeks 7‐8 (CS20) via immunofluorescence, followed by the detection of circulating αβ and γδ T cells of thymic origin at weeks 11‐12.^10^, ^31^, ^32^, ^33^, ^34^, ^35^ Haddad et al^33^ described a population of thymic‐homing CD45^+^CD7^+^ cells arising from the fetal BM at week 8 of gestation, which may be the source of a second wave of thymic colonizing cells. On the contrary, scRNA‐seq data showed that early thymic progenitors at gestational week 8 share a similar transcriptomic profile with FL IL7R^+^ cells, raising the possibility that FL progenitors are responsible for the second wave of thymic colonization.^9^ The definitive HSC arises in the AGM at CS14‐16 (mid‐week 5) and migrates to the FL at CS16‐17 (week 6), where the FL is also populated by YS‐derived hematopoietic progenitors.^4^, ^36^ The emergence of T cell progenitors concurrently with HSCs at the CS16 AGM and their presence in the CS17 FL (approximately week 6) has been reported previously.^9^, ^36^ As such, these observations are consistent with a scenario in which non‐HSC AGM‐derived T cell progenitors, or more primitive yolk sac derived precursors already present in the fetal liver at week 6, are the earliest thymic seeding cells.

T cells developed from YS or AGM hematopoiesis also have different TCR repertoires. Experiments in the mouse system by Yoshimoto et al^7^ showed that YS cells could give rise to a large proportion of cells bearing αβ TCRs with some Vβ chain variability. However, there was a distinct difference in Vβ chain usage between αβ T cells derived from the E9.5 YS and AGM. While YS‐derived αβ T cells were biased towards the use of Vβ8, AGM cells showed a similar distribution of Vβ chain usage as adult splenic αβ T cells^7^ Moreover, the ability of the YS EMP program to give rise to αβ T cells is supported by in vitro human iPSC differentiation experiments, which showed that after prolonged co‐culture on OP9‐DL4 cells, αβ T cell populations became the dominant subset.^21^ This result is consistent with the observation that compared to γδ T cells, αβ T cells predominate in the fetal and adult thymus.^10^ However, unlike YS derived γδ T cells that occupy the mucosa and self‐renew throughout life without being replaced by thymic emigrants,^37^ it is unclear whether YS‐derived αβ T cells persist into adulthood and whether they are subsequently replaced by αβ T cells whose origins can be ultimately traced back to the AGM.

### 3.2 RAGs in early embryonic T cell development

T cell development is a highly coordinated process in which stage‐specific expression of the RAG1 and RAG2 genes marks key junctures (Figures 3 and 4). Mice lacking RAG1 or RAG2 fail to initiate V(D)J recombination, resulting in the arrest of T cell development at the CD4^−^CD8^−^ double negative (DN) stage.^38^, ^39^ In both mice and humans, RAG genes are expressed in two separate waves, resulting in the temporal regulation of recombination at different TCR loci. During the first RAG expression wave, TCR rearrangement occurs at the TCR β, γ and δ loci during the DN stage.^40^ Successful rearrangement of the γ and δ TCR loci allows for the formation of a γδ TCR, whereas a successfully rearranged β TCR will pair with the invariant pre‐TCRα to form the pre‐TCR complex.^40^ Subsequent signaling via the γδ TCR or pre‐TCR complex initiates a negative feedback loop, inhibiting RAG1 and RAG2 transcription, resulting in the cessation of RAG expression at the late DN stage.^41^, ^42^ This first wave of RAG expression is likely to presage the appearance of γδ T cells that have been observed as early as week 6 in the FL, suggesting the existence of extrathymic RAG expression during embryonic T cell development.^31^ Indeed, investigations using a Rag1:GFP mouse strain identified that the first thymic seeding cells are Rag1^+^.^19^ Although the developmental origin of these cells is unknown, it is noteworthy that the AGM and the FL maintain high levels of Notch ligand expression by the arterial endothelial cells,^4^ potentially providing an environment conducive for lymphoid priming.

As noted above, it is likely that RAG recombination of the TCR locus is not strictly limited to the thymus. Evidence for thymus‐independent T cell lymphopoiesis is available from studies using RAG2‐GFP reporter athymic mice. Guy‐Grand et al^43^ showed that in the absence of the thymus, RAG2 and RAG1 expression can be found in cells of the mesenteric lymph node, cell that potentially give rise to a population of intraepithelial lymphocytes (IEL). The mouse IEL compartment is ontogenically diverse, with the γδ TCR subset biased towards the expression of a Vγ7^+^ TCR and CD8αα.^44^ This subset of IELs has pre‐programmed effector functions and releases TNF‐α and IFN‐γ rapidly after activation—a trait shared with other innate‐like T cells.^45^ In the absence of the thymus, mesenteric T lymphopoiesis is biased to produce γδ T cells, perhaps a result of the lack of appropriate developmental signals required for αβ TCR recombination.^43^

Notwithstanding findings from perturbation studies, current evidence indicates that the development of αβ T cells in vivo is thymus dependent. Unlike γδ T cells, which only require the first wave of RAG activation for their development, αβ T cells are generated only after the second wave of RAG expression. This second, more intensive wave of RAG expression occurs at the CD4^+^CD8^+^ double positive (DP) stage of thymocyte development, where RAG‐mediated recombination is restricted to the TCRα locus^46^ (Figure 3). Productive rearrangement results in the formation of the αβ‐TCR complex and its expression on the thymocyte cell surface—setting the stage for TCR‐mediated positive selection.^47^ The intricacies of epigenetic factors that control the two waves of RAG expression in thymocytes has been exhaustively studied and reviewed elsewhere.^48^ However, it is important to further explore the role of RAG expression levels, dynamics, and kinetics in cell fate determination, in the context of embryonic lymphoid differentiation.

Several lines of evidence suggest that hematopoietic progenitors from different ontogenetic waves have different characteristics that reflect their proliferative capacity and embryonic location, as well as lymphoid lineage potential. For example, YS hematopoiesis does not require expression of the proto‐oncogene, c‐Myb, nor upregulation of genes belonging to HOXA cluster, the latter being involved in establishing embryonic axial patterning but and also playing a role in cell proliferation.^15^, ^49^

RAG2 protein expression is highly cell cycle dependent, where it accumulates at the G1 phase and is degraded prior to entering the S‐phase.^50^ This CDK‐dependent degradation of RAG2 also links RAG1 to the cell cycle, as RAG1 self‐aggregates to form an inactive nuclear complex in the absence of RAG2.^50^, ^51^ Epigenetic targeting of the RAG complex is a highly ordered process in which different TCR loci are targeted at different stages of thymocyte development.^52^ RAG2 is responsible for recruiting the recombinase complex to active chromatin via its binding to trimethylated H3K4.^53^ This recruitment allows the recombinase complex to bind to the RSS sequences flanking J genes, initiating V(D)J recombination. In thymocytes, early opening of the γ and δ loci is dependent on IL7‐STAT5 signaling, coinciding with the high expression of the IL7 receptor on DN thymocytes.^54^, ^55^ Therefore, cell cycle regulation in uncommitted progenitors at different embryonic development stages may impact the gene regulatory networks that control RAG gene expression, and hence constrain the differentiation potential of their progeny. These observations also give pause for inferring gene activity from RNA‐seq data and argue for using functional readouts, such as TCR rearrangement, as additional measures of RAG activity.

### 3.3 Innate lymphoid cells

Innate lymphoid cells are a group of cells with characteristics that mirror adaptive T cell subsets but lack TCRs. For example, NK cells share properties with cytotoxic CD8^+^ T cells, while ILC group 1 (ILC1) cells have similarities with Th1 cells, ILC2 cells with Th2 cells, and ILC3 with Th17 cells, which have been systemically reviewed elsewhere^56^ and in the review by Van de Pavert in this volume. Recently, investigations into ILC and T cell development suggest that ILCs belong to a branch of the lymphoid lineages that are first observed during FL hematopoiesis from weeks 7‐8 of human development, and which is distinct from a distinct branch that gives rise to B cells.^5^ Nevertheless, a non‐functional RAG‐mediated rearrangement history has been documented at TCR loci of ILC2s,^57^ suggesting that ILC and T cells share a common ancestry that persists up until RAG gene activation. Interestingly, there is evidence that NK cells that have a history of RAG expression develop a distinct memory‐like phenotype following cytomegalovirus infection, while NK cells that have never expressed RAG genes are more prone to apoptosis and are less efficient at DNA repair^58^, ^59^ (Figure 2). These studies suggest that expression of RAG genes may represent an important branch point in the developmental pathways giving rise to conventional T cells and different subclasses of ILC/NK cells.

As with T cell differentiation, much of our knowledge of ILC development has focused on their genesis from mouse BM‐HSCs, with limited reports describing their ontogeny during embryogenesis. For NK cells, mouse YS cells isolated from E9.5 can effectively generate CD3ε^−^CD19^−^CD122^+^ NK1.1^+^ NK cells when co‐cultured with OP9 cells in vitro, consistent with previous reports showing lymphoid potential of E9.5 YS cells.^60^ In the mouse embryo, NK1.1^+^ NK cells are not detectable until E13.5 in the FL. Additionally, conditional lineage tracing experiments using Csf1r^MerCreMer^Rosa26^YFP^ mice confirmed that FL NK cells at E13.5‐15.5 are derived from YS EMPs labeled at E9.5. However, for lymphoid tissue inducer (LTi) cells, another type of ILC, lineage tracing experiments using Cdh5‐CreERT2^+/−^;Rosa^tdT^ and Cxcr4‐CreERT2+/;Rosa^tdT^ showed that LTi cells are derived from hemogenic endothelial cells of the AGM instead of the YS.^61^ In contrast to the NK lineages, there is no evidence to suggest that RAG genes are expressed in ILC precursors, potentially signifying a distinction between the pathways giving rise to different innate cell types derived from lymphoid committed progenitors.

### 3.4 B cells

B lineage cells give rise to antibody producing cells via process that is critically dependent on the expression of RAG genes during the early stages of B cell differentiation. B cells have been classified as belonging to one of two subgroups, B1 and B2, with the latter being the predominant type in the adult that provides adaptive immune functions.^62^ In mice, B1 cells have an innate‐like phenotype and produce antibodies that target common pathogens and low affinity antibodies that recognize self‐antigens^63^ (Figure 3). The fetal ontogeny of innate‐like B1 cells has been studied extensively in mice, and this literature is reviewed in two additional chapters in this edition of *Immunological Reviews*. B2 cells are the “conventional” B cell subset, showing a high diversity of B cell receptors and giving rise to antibody‐secreting plasma cells following activation by foreign antigens, a process that is critically dependent on T cell help. It has been well established that B2 cells develop postnatally from adult BM HSCs and further mature into marginal zone or follicular B cells in secondary lymphoid organs.^62^

In mice, B1 cells can be further categorized into CD5^+^ B1a and CD5^−^ B1b cells. Transplantation of adult BM‐ or FL‐derived HSCs into immunodeficient mice was able to generate B2 cells and B1b cells, but not B1a cells,^1^, ^2^ suggesting that B1a cells arise from a fetal progenitor that lacks HSC‐like activity. Consistent with this hypothesis, B1 cells could be generated from YS and AGM progenitors at approximately E9 (prior to HSC specification) and then subsequently persisted throughout adulthood.^6^, ^64^ Consistent with these findings, a lineage tracing studies of adult HSCs using Pdzk1ip1‐CreER:Rosa26^TdTomato^ mice showed that labelled HSCs made a limited contribution to the total B1a cell pool of adults.^65^ Similarly, Hadland et al^66^ identified a common precursor for both B1a and B2 cells at E9.5, prior to the generation of HSCs.

However, there are contrasting findings experiments where FL HSCs and adult BM have been examined for their capacity to generate B1a cells.^67^, ^68^, ^69^ In adult Rag1 deficient mice, functional rescue of B cell‐specific Rag1 expression permitted the generation of B1a‐like cells that colonized the peritoneal cavity.^67^ Similarly, Kristiansen et al^69^ used cellular barcoding to show FL HSCs were indeed capable of giving rise to both B1a and B2 cells. In addition, LIN28B, an RNA binding protein whose expression diminishes after birth, has been found to be associated with the fetal ontogeny of B1a cells.^69^ Indeed, recent scRNA‐seq analysis showed that the other LIN28 paralog, LIN28A, is restricted to early primitive hematopoietic progenitors in humans.^3^ These findings indicate that B1a cells arise in multiple waves of hematopoiesis. During early embryonic development, these waves include fetal non‐HSC hematopoietic progenitors and subsequently fetal and adult HSCs. Interestingly, fetal HSCs appear to be more efficient at producing B1a cells compared adult HSCs, a developmental switch in B lymphopoiesis potentially controlled by LIN28B.^69^, ^70^, ^71^ These more recent findings are consistent with earlier studies showing the capacity of adult HSCs and common lymphoid progenitors to generate B1 cells is diminished relative to their neonatal counterparts^72^ and reflect foundational work demonstrating the clear differences between the development trajectories of fetal vs adult derived B cells.^73^, ^74^

The extensive study of B1 cells in mice raises the question as to whether there is a human counterpart of this B cell subset. Although the existence of human B1 cells has not been firmly established, Griffin et al^75^ identified a B cell population in human umbilical cord blood and adult peripheral blood with surface phenotype of CD20^+^CD27^+^CD43^+^CD70^−^, which showed a similar function to mouse B1 cells in their ability to spontaneously produce IgM. Nevertheless, understanding the emergence of B1 and B2 cells during human fetal B cell development remains to be investigated.

In humans, B‐lineage cells have been found in the FL as early as week 7 and in the fetal BM by week 11.^5^, ^76^ O'Bryen et al identified a CD10 negative prepro‐B progenitor in the FL at week 7, potentially representing the earliest B cell progenitor downstream of the early lymphoid progenitor (ELP). This population showed a comparable gene expression profile to adult prepro‐B progenitors, with the exception that fetal prepro‐B progenitors had higher expression levels of RAG1, which is important for B cell receptor (BCR) rearrangement, and of DNTT, which has a key role in the generation of BCR diversity.^77^ Whether this progenitor arose from a non‐HSC precursor is unclear, appearing in the FL 1 to 2 weeks following the generation of HSCs in the AGM.

### 3.5 RAG activity during B cell development

Variation in the level of RAG expression during fetal vs adult B cell development has not been extensively investigated. It is known that mice lacking a functional RAG1 gene fail to generate an adaptive immune cell repertoire, with B cell development arrested at the pro‐B cell stage.^78^, ^79^ In humans, mutations in RAG1 and RAG2 have been found in individuals with severe combined immunodeficiency (SCID), Omenn Syndrome, and combined immune deficiency with granulomas or autoimmunity (CID‐G/AI) (reviewed in 80). Dependent on the extent to which RAG activity is affected, these individuals sometimes exhibited high levels of autoantibodies and lack T and B cell receptor diversity.^81^, ^82^

Paralleling observations concerning T cell differentiation, RAG activity can be observed as two waves during B cell development in the BM (Figure 4). The first wave of RAG expression correlates with rearrangement of the Ig heavy chain (IgH) locus while the second coincides with rearrangement of the Ig light chain (IgL) locus. RAG expression is only fully inactivated when immature B cells progress to become mature B cells which co‐express sIgD.^83^ In addition to these processes that are clearly linked to B cell development, there is some suggestion that RAG activity may have a function in peripheral B cells. Studies have shown that B cells can reactivate RAG expression in germinal centers (GC) and human tonsils for a secondary gene rearrangement known as receptor revision.^84^, ^85^, ^86^ Indeed, these additional sites of RAG1 expression pose an additional hurdle to clearly determining whether different levels of RAG1 expression are a characteristic of cells arising from distinct developmental trajectories.

## 4 LYMPHOID DIFFERENTIATION FROM HUMAN PLURIPOTENT STEM CELLS

### 4.1 General methodology of PSC‐based hematopoiesis

The concept underlying the differentiation of lymphoid cells from PSCs is to make hematopoietic progenitor cells and then to induce lineage specification based on methods previously established with cord blood or BM derived HSPCs. Initial studies of PSC differentiation showed that CD34^+^ hematopoietic cells could be co‐cultured with mouse YS‐derived endothelial cells and BM cells in the presence of fetal bovine serum.^87^ Since this pioneering work, PSC differentiation protocols have developed in complexity and sophistication, providing the opportunity to not only generate clinically important cell types, but to also understand the mechanisms that govern the differentiation process itself.

Expression of key transcription factors distinguishes different waves of hematopoiesis that have differential lymphoid potential. In mice, Sox17 marks the arterial endothelial cells of the AGM region as well as emerging HSCs, and conditional deletion of Sox17 driven by Tie2‐Cre or Mx1‐Cre severely disturbs fetal hematopoiesis, blocking HSC development.^88^, ^89^ In Sox17:GFP mice, hemogenic endothelium and emerging HSCs of the AGM expressed GFP, a conclusion consistent with in vitro differentiation experiments showing mouse ESC‐derived hemogenic endothelium also expressed Sox17.^90^ Similarly, differentiation experiments using a human SOX17:mCHERRY reporter ESC line showed that SOX17^+^ vasculature, which had a gene expression profile resembling vessels of human AGM, was able to generate lymphoid cells marked by the expression of CD7.^12^, ^49^ Interestingly, although Sox17 is pivotal for the definitive AGM hematopoiesis in vitro and in vivo, mouse ESCs deficient for Sox17 are still competent to generate blood cells, but only those representing the myeloid lineages.^90^, ^91^

Since yolk sac like blood cell progenitors yield predominantly erythroid and myeloid lineages, robust T lymphoid potential has often been regarded as a critical feature of definitive blood progenitors. Studies of in vitro mesoderm patterning indicated that TGF‐β signaling inhibition or a WNT agonist at this stage increased the yield of CD34^+^CD43^+^ hematopoietic progenitors, and these blood cells exhibited robust capacity to form CD5^+^CD7^+^ T cell progenitors when co‐cultured with OP9‐DLL4 mouse stromal cells^25^, ^92^, ^93^ (reviewed in detail below). These findings are consistent with the observation that inhibition of TGF‐β signaling and/or WNT pathway activation generates SOX17^+^ hemogenic vasculature that produces definitive blood cells, including lymphoid cells identified by RAG1:GFP reporter PSC lines.^12^, ^49^ However, as noted in the prior discussion concerning lymphoid development in vivo, the production of lymphoid cells per se does not provide evidence for intra vs extra embryonic‐like origins of the lymphoid precursors. In the context of in vitro studies, such origins can only be inferred on the basis of a developmental history that includes the expression of HOXA cluster genes.^4^ Nevertheless, despite uncertainties surrounding the ontogenetic pathways these cells represent, PSC‐based hematopoiesis has laid the foundations for the generation of lymphoid cells in vitro, providing a platform for modelling development and creating future cell‐based immunotherapies.

### 4.2 T cells: Engineered murine stromal cells expressing NOTCH ligands

**FIGURE 5:**
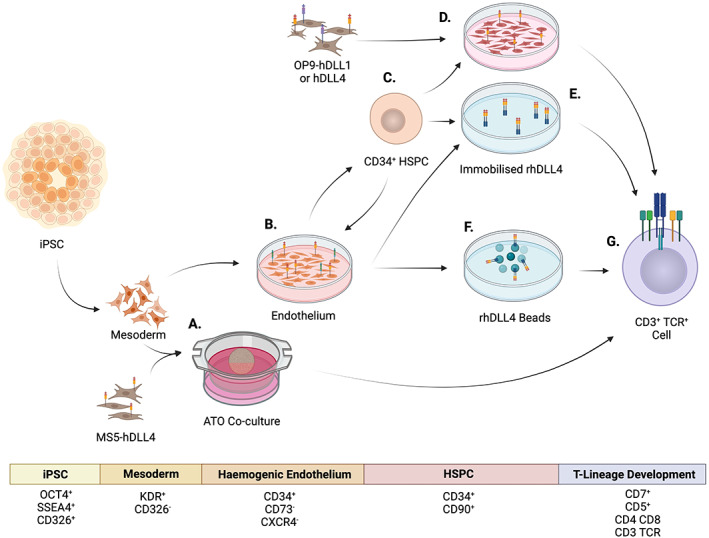
Hematopoietic and lymphoid differentiation of PSCs to T cells in vitro. At the mesodermal stage, cells are either co‐cultured with human DLL4 (hDLL4)‐expressing MS5 cells to form artificial thymus organoids (ATOs) (A) that produced CD3^+^TCR^+^ T cells (G), or directly differentiated to hemogenic endothelial cells (B). Hemogenic endothelial cells can be further differentiated to CD34^+^ hematopoietic stem/progenitor cells (HSPCs) (C). Co‐culture of HSPCs with hDLL1/4 OP9 monolayer (D) is also able to generate T cells. Alternatively, including immobilized recombinant human DLL4 (rhDLL4) (E) or rhDLL4 beads (F) in cultures of hemogenic endothelial cell or HSPCs is able to generate T cells. Expression kinetics of key cell surface markers during iPSC‐to‐T cell differentiation is listed in the table inset (Figure created with BioRender.com).

Based on earlier experiments performed with adult or cord blood derived CD34^+^ cells, one of the most robust ways of generating T lineage cells in vitro is to co‐culture PSC‐derived hematopoietic progenitor cells with mouse stromal cells engineered to overexpress Notch ligands (Figure 5). Initially, stromal cell lines such as OP9 and MS5, derived from mouse bone marrow, were used to support B lymphopoiesis in vitro.^94^ Subsequently, these lines were engineered to express Notch ligands, enabling them to support T cell differentiation^94^, ^95^, ^96^ For example, OP9 cells expressing Dll1 ligands support the differentiation of mouse FL, BM and cord blood‐derived HSPCs to T cell precursors expressing either the γδ or αβ TCR and the T cell surface marker CD3.^95^, ^97^ In addition to DLL1, versions of these lines expressing other NOTCH ligands have been derived for studying lymphoid differentiation.^98^, ^99^ As an alternative to OP9, the mouse BM derived MS5 stromal line has been also used for the overexpression of Notch ligands to support of T cell differentiation. Aggregating DLL1‐expressing MS5 stromal cells with human cord blood HSPCs on an air‐liquid interface created a 3D T cell‐permissive environment, termed an artificial thymic organoid (ATO).^100^ In the ATOs, the DLL1‐expressing MS5 cell lines can efficiently support the development of TCR^+^CD3^+^ as well as CD8 single positive cells, with 20%‐40% of cells in culture expressing TCR and CD3 after 6 weeks of culture. These mouse stromal cell‐based systems have been adapted to direct the differentiation of human PSCs to the T cell lineage.^24^, ^25^

Reflecting the origins of the OP9 based T cell induction systems, differentiation of human PSCs to T cells on the OP9‐Notch stromal cells commonly required the generation of hematopoietic progenitors prior to their addition to stromal cell co‐cultures.^101^, ^102^ However, further refinements of the ATO system have incorporated mesoderm induction and hematopoietic specification stages into the workflow, greatly simplifying the generation of lymphoid cells while simultaneously increasing the efficiency of T cell production.^100^ Indeed, the efficiency of the ATO system is a substantial improvement over the OP9‐based monolayer systems; the 3D microenvironment inherent to the ATO delivers more robust and efficient outcomes. This is possibly because it enables high cell densities that are a characteristic of thymopoiesis. Nevertheless, the reliance on mouse stromal cells by both monolayer and ATO cultures makes dissection of the differentiation pathways from PSCs to T cells less experimentally tractable.

Despite the successes of current in vitro lymphoid differentiation methods, understanding how these systems enable cells to traverse the many complex steps conventionally associated with T cell development in vivo remains a challenge. In the thymus, developing T cells undergo positive selection as they rearrange their TCR genes and interact with HLAs expressed by thymic epithelial cells and thymic resident APCs.^103^ In the human thymus, thymocytes express MHC I that can promote selection of CD8^+^ single positive (SP) cells through thymocyte‐thymocyte interactions.^104^, ^105^ This mechanism of selection is mirrored in in vitro differentiation systems that preferentially generate CD8^+^ SP cells and are less efficient at producing CD4^+^ SP cells, presumably due to the lack of MHC II‐expressing cells.^106^, ^107^ Nevertheless, the ATO system does allow the generation of CD4^+^ thymocytes after extended culture periods, and both the CD8 and CD4 SP T cells upregulate CD25 and proliferate in response to anti‐CD3 activation, suggesting further maturation.^24^, ^100^ Leveraging the efficiency of this system, recent experiments have employed ATOs to model human T cell lymphopenia and as a platform to study gene therapy options for severe combined immunodeficiency (SCID).^108^, ^109^

An alternative organoid system enables the generation of RAG1^+^ cells directly from PSC‐derived hemogenic vasculature in vitro.^12^ In this system, DLL4^+^ endothelial cells within hemogenic vasculature enable the emerging hematopoietic progenitors to towards the T cell lineage in the presence of exogenous IL‐7. Nevertheless, the generation of CD3^+^ cells in this system was limited and could only be achieved by transferring cells onto OP9‐DLL4 stroma. As such, the limitations of this specific organoid system to support T cell differentiation possibly reflected suboptimal access of developing cells to Notch ligands that are required for both expression of RAG genes and T cell development.^110^

### 4.3 T cells: Immobilized recombinant Notch ligands

Because of their defined nature, stromal‐free systems that rely on recombinant human DLL4 proteins to drive T cell differentiation are potentially more suitable for the generation of cell types that might be utilized in the clinic.^111^, ^112^, ^113^ Recombinant DLL4 has been shown to efficiently promote T cell differentiation from human iPSCs reprogrammed from T cells, known as T‐iPSCs.^114^ In the study of Iriguchi e al, hemogenic endothelium‐containing embryoid bodies that were seeded onto plates coated with immobilized DLL4 generated functional TCR‐αβ^+^CD3^+^CD8^+^ T cells and functional chimeric antigen receptor (CAR)‐T cells. Three additional groups reported success in generating TCR^+^CD3^+^ cells using recombinant DLL4, albeit provided using different physical configurations.^115^, ^116^, ^117^ Trotman‐Grant et al showed that both plate‐bound and microbead‐bound DLL4 promoted T‐lineage differentiation from iPSC‐derived endothelial cells with microbead‐bound DLL4 being more adaptable for upscaling. In addition to DLL4, Jing et al and Michaels et al showed that the addition of VCAM1, an adhesion molecule highly expressed in the thymic cortex, increased the frequency of T cell‐biased hematopoietic progenitors during the hematopoietic to endothelial transition stage. Jing et al also identified the polycomb group protein EZH1 as an important repressor of T cell fate entry, acting via the silencing of early lymphoid genes, including the Notch‐signaling related protein, HES1.

Although these groups have generated cytotoxic CD8^+^ T cells, it is also notable that none reported the efficient generation of CD4^+^ T cells, paralleling results with other in vitro systems that deliver exogenous Notch ligands. All four groups reported successful generation of TCR‐αβ CD4^+^CD8β^+^ DP cells, and three groups induced further maturation in vitro using either anti‐CD3 alone or in combination with anti‐CD28 in the presence of different cytokine combinations.^114^, ^115^, ^116^ However, Trotman‐Grant et al^117^ showed that pro‐T cells generated from their protocol were able to engraft murine thymi and differentiate into CD4 SP cells, demonstrating that these cells retain CD4 potential. Paralleling results of studies using stromal based systems, cytotoxic T cells produced using immobilized DLL4 show functional maturity, evident by their proliferation and production of TNF‐α and IFN‐γ in response to anti‐CD3 or PMA/Ionomycin.^114^, ^115^, ^116^, ^117^

### 4.4 T cells: Chimeric antigen receptor‐T cells from PSCs

One of the most exciting prospects to come out of the development of in vitro T cell differentiation protocols is the potential to produce iPSC‐derived CAR‐T cells. CAR‐T cell designs often feature the expression of a short chain variable fragment (scFv) against a specific antigen as an activation receptor in lieu of their CD3‐TCR complex (reviewed in 118). Newer generation CARs also feature scFvs fused with the intracellular domains of CD28 (or 4‐1BB) as well as the CD3ζ chain to facilitate CD3‐independent signal transduction.^118^, ^119^ As mentioned previously, most in vitro differentiation protocols are biased towards the differentiation of CD8 SP cells, a phenotype that is highly desirable for the cytotoxic potential required for most CAR‐T designs. As autologous iPSCs can be easily expanded in vitro, the production of clinically relevant numbers of CAR‐T cells can be achieved more easily compared to expanding autologous T cells.^120^

Human iPSCs have been used to generate CAR‐T cells which target cells expressing the B‐lymphoma antigen CD19.^121^ Cells differentiated using the monolayer OP9‐DL1 system generated CAR‐T cells with an innate‐like phenotype, releasing TNF‐α, Il‐2, and IFN‐γ when co‐cultured with CD19‐expressing cells. These iPSC‐derived CAR T cells could be expanded in vitro and retained their cytotoxic function after multiple rounds of expansion. More recently, anti‐CD19 CAR‐T cells have also been developed using both the MS5‐DLL4 ATO system and using immobilized recombinant DLL4.^109^, ^114^, ^115^ Compared with autologous PBMC‐derived CAR‐T, iPSC‐derived T cells generated using these two protocols show lower expression of the exhaustion markers PD1 and CTLA4, but higher expression of the immune checkpoint molecule LAG3.^109^, ^114^ Both of these protocols produce CAR‐T cells with intact cytotoxic functions, with CD19‐specific cell induced proliferation and cytokine production. Using mouse xenograft models, iCAR‐T cells produced show comparable efficacy to conventional CAR‐T cells. However, one of the limitations of xenograft models for assessing the function of in vitro derived T cells is that many mouse cytokines do not bind or activate their cognate receptors on human cells. Experiments indicate that the in vivo efficacy of ATO‐derived iCAR‐T cells could be enhanced via a co‐injection of mice with human IL‐15‐producing nurse cells.^109^

In addition to efforts to create cells based on the archetypical TCR‐αβ T cell phenotype, research is also exploring the generation of mucosal associated innate T cells (MAIT) and NKT CAR‐T cells, as well as γδ T cells.^122^ In humans, the most abundant subtype of γδ T cells expressed a TCR containing the Vγ9δ2 segment. These cells represent a promising platform for CAR‐T derivation due to their potent cytotoxic capabilities, recognition of phosphoantigens in malignant cells via their γδ‐TCR, as well as their MHC‐independence.^123^, ^124^ Although the development of γδT‐iPSCs has been reported recently, their redifferentiation into functional γδ CAR‐T cells is yet to be established.^125^

### 4.5 TCR repertoire of PSC‐derived T cells

It has been known that prenatal T cells possess TCRs with a shorter CDR3 length due to a lower level of DNTT expression, resulting in fewer N‐nucleotide additions.^126^ Similarly, adult peripheral blood T cells and adult thymocytes have TCRs with longer CDR3 segments compared to cord blood‐derived T cells.^116^, ^127^ ATO‐derived T cells from a hESC source also show shorter CDR3 length compared to primary postnatal thymic and peripheral blood T cells, showing limited TdT activity within developing lymphoid progenitors in hESC‐ATOs. This is consistent with the idea that hESC‐ATO‐derived T cells are more closely related to those generated during fetal thymic T lymphopoiesis.^24^ Likewise, T cells derived from iPSCs differentiated using immobilized DLL4 have TCRs with CDR3 segment lengths comparable to cord blood‐derived T cells, but shorter than T cells derived from adult HSPCs.^116^ Interestingly, Jing et al^115^ showed that knock down of the gene encoding the histone methyltransferase EZH1 resulted in T cells with a more adult‐like TCR phenotype, with longer CDR3 segments, potentially reflecting elevated expression DNTT.

The monolayer OP9‐DLL4 system has been shown to produce populations of T cells with a highly diverse TCR repertoire at the protein level.^128^ The TCR‐β repertoire of naive T cells of young adults and iPSC‐derived T cells using OP9 monolayers both show a prevalence of Vβ chains such as TRBV7, 12, 5, and 18. Similarly, T cells created using the ATO system show TCR repertoire diversity that includes TCR‐β variable chain usage that has parallels with that seen in with primary thymocytes.^24^, ^100^ Notably, TRBV5.1, 7.1, 2, 8, and 14 are commonly used, similar to T cells differentiated using the OP9 monolayer system^128^ Collectively, these observations indicate that the relationship between the complexity of the TCR repertoire and different differentiation trajectories is not clear cut, with in vitro systems creating cells with repertoires that cannot easily be equated with specific waves or populations of developing T cells in vivo.

T cells derived from recombinant Notch ligand cultures also showed highly diverse TCR‐β chain usage. In comparison with cord blood‐derived T cells, adult peripheral blood and adult thymic TCR‐β repertoires, T cells generated by Michaels et al^116^ possessed TCRs with a higher prevalence of TRBV21, 23 and 24 segments and a reduced frequency of TCRs containing the TRBV28 segment. Consistent with findings related to the length of the CDR3 segment in T cells derived in the context of EZH1 knockdown, Jing et al^115^ found EZH1 deficient T cells had a TCR repertoire with greater similarity to cord blood and adult T cells. Whether this difference is a result of protocol differences or due to EZH1 KD resulting in a TCR repertoire that is more similar to postnatal sources is unknown. Comparing δ‐chain preference between cord blood‐derived and iPSC‐derived T cells, Michaels et al^116^ also showed that iPSC‐derived T cells derived on recombinant DLL4 have a bias towards δ2 chain usage. This result is similar to δ‐chain preference seen from monolayer OP9 systems using prenatal sources of HSPCs.^129^

### 4.6 NK cells

Although there are multiple protocols to generate NK cells from human PSCs, the investigation of other innate lymphoid lineages has been limited. Furthermore, unlike the generation of these cell types from HSCs in vitro, the developmental pathways directing PSCs to NK cells and ILCs are poorly defined, possibly because there are multiple waves of hematopoietic development that could potentially contribute to their genesis^60^, ^61^ Early strategies for creation of innate lymphoid cells co‐cultured HSPCs with mouse OP9 stromal cells in medium supplemented with cytokines, such as IL3, IL7, IL15, FLT3‐ligand, and SCF.^130^ IL3, also known as multilineage colony stimulating factor, is often used during the first week of differentiation, potentially to support the expansion of multipotent progenitor populations. IL7 is an important lymphoid factor that supports the differentiation of B cells, T cells, and NK cells from multipotent hematopoietic progenitor cells and is indispensable for T lymphoid development. IL15, which shares components of its receptor with IL7, is known to be critical for the specification of NK cells from committed lymphoid progenitor cells and for the ongoing survival and function of this cell type.^131^

Protocols that generate YS‐like EMPs from PSCs can be co‐opted to produce NK cells.^60^, ^132^ These cells arise via a pathway that is distinct from the classical lymphoid differentiation pathway that begins with HSCs in the adult bone marrow. Using a “spin embryoid body” approach that produces YS‐like EMPs,^133^ hematopoietic progenitors could be differentiated to cytotoxic NK cells and stromal cell components inherent in PSC‐derived embryoid bodies could facilitate NK cell maturation, mitigating the requirement for mouse OP9 cells.^132^, ^134^ Recently, Dege et al^60^ have leveraged knowledge concerning the distinct waves of hematopoiesis to examine the origins and properties of NK cells emerging from primitive and definitive hematopoiesis using PSC differentiation. Previous experiments indicated that manipulation of the WNT signaling pathway could be used to direct PSC differentiation to either the YS‐like or embryo‐like hematopoiesis.^49^, ^92^ Using this knowledge, Dege et al^60^ were able to show that both waves of hematopoiesis were able generate NK cells, but these NK cells had distinct characteristics. NK cells emerging from a YS‐like EMP progenitor displayed potent cytotoxic functions, a characteristic conventionally associated with CD56‐low NK cells from in vivo sources.^135^ Conversely, NK cells emerging from a “embryonic‐like” wave of blood cell development showed higher expression of effector molecules such as IFN‐gamma, a property shared with CD56‐high NK cells.^136^ Consistent with these findings, PSC‐derived NK cells with a putative YS‐like origin showed robust anti‐tumor activity, a characteristic that could underpin new NK cell therapies in combination with CAR technologies.^132^ Nevertheless, despite potential insights into the origin of these two distinct NK cell types, it is still unclear how the ontogeny of either related to the classical lymphoid differentiation pathways normally associated NK development.

### 4.7 B cells

Similar to T cell generation, B cell differentiation protocols from human PSCs are based on experiments initially performed with CD34^+^ human cord blood cells. One such early study incorporated experiments in which cord blood‐derived CD34^+^ HSPCs were transduced with a lentiviral construct encoding a monoclonal antibody recognizing an HIV‐1 antigen.^137^ In order to induce B cell activation, pre‐B cells were co‐cultured on a modified MS5 stromal cells expressing human CD40L to simulate T cell‐dependent B cell activation. In addition to CD40L, developing B cells were provided with IL‐2, IL‐10, and CpG DNA, signals required by naive B cells for antigen‐independent B cell activation. These culture manipulations, in conjunction with lentiviral genetic modification, enabled B cell progenitors to be matured into functional antibody‐secreting plasma cells (approximately 20%).

Paralleling the development of T cell differentiation protocols, B cell differentiation from PSCs can be separated into two distinct phases. The first phase involves the generation and enrichment of CD34^+^ progenitor cells, creating a starting population that is a surrogate for CD34^+^ cells derived from sources such as cord blood. It should be noted that although these CD34^+^ cells have multilineage potential, they do not contain cells with HSC‐like repopulating potential. Beginning with this population, the second phase follows a roadmap for in vitro of B cell differentiation established using in vivo derived CD34^+^ cells. As such, CD34^+^ cells are co‐cultured with MS5 stromal cells in presence of cytokines and growth factors favorable for B lineage differentiation.^138^, ^139^, ^140^, ^141^ These factors commonly include stem cell factor (SCF), FMS‐like tyrosine kinase 3 ligand (Flt3‐L), interleukin‐3 (IL‐3), factors that could theoretically support expansion of progenitor pool, and interleukin‐7 (IL‐7), a key factor for lymphoid development. However, B cell differentiation from human PSCs typically generates cells corresponding to the pre‐B cell stage, marked by expression of CD19, either intracellular or low‐level surface expression of IgM and the absence of surface IgD.^142^ Further differentiation to functional B cells has only been possible following genetic modification or co‐culture with MS5 stromal cells for extended periods (5‐8 weeks instead of 2 weeks).^137^ Indeed, at the end of differentiation, CD45^+^CD19^+^CD10^+^ cells were observed, albeit at low frequency (<10%). This indicates that for most current protocols, differentiation is terminated at an “immature” state roughly equating to the pre‐B cell stage, shown by the expression of the surrogate light chains, VpreB (? VPREB1) and λ‐like (?IGLL) with minor expression of surface IgM.

The complex requirements for B cell development underlie the many challenges facing efforts to generate B cells in vitro. Developmentally, plasma B2 cells are thought to arise from the definitive intraembryonic wave of hematopoiesis that gives rise to HSCs.^3^, ^4^, ^5^, ^23^ The fact that HSC generation from PSCs has not been achieved in the absence of genetic modification raises the possibility that differentiation protocols have also not yet produced precursors with appropriate B cell potential. Even if this hurdle can be cleared, in vitro systems that can generate plasma cells capable of producing high affinity antibodies will require the development of artificial systems that provide an environment that will enable affinity maturation.

## 5 SUMMARY AND CHALLENGES

Lymphoid cells are derived from the latter two waves of embryonic hematopoiesis. Research using mouse models and human tissues indicates that the first cells with lymphoid potential originate from definitive hematopoiesis occurring in the YS, independent of the long‐term HSCs. Mouse studies have also proven that YS progenitors are able to generate all typical lymphoid lineages, including T cells, B cells, and NK cells, although the differentiation of hematopoietic progenitors to functional lymphoid cells occurs in the embryo proper. Despite the identification of IL7R^+^ cells representing early lymphoid cells in the human YS, there lacks direct functional evidence to show human YS progenitors do give rise to T cells and/or B cells. This question has been partially addressed using differentiation of human PSCs in vitro, where PSC‐derived YS‐like progenitors have been shown to produce T cells; however, unequivocal correspondence between PSC‐derived YS‐like progenitors and bona fide human YS progenitors will require further research. As such, it is difficult to definitively fate‐map lymphoid cells in the adult back to their developmental origin, a situation that presents challenges for generating functional blood cells in vitro and for investigating the developmental origin of hematopoietic malignancy.

Nevertheless, protocols for the differentiation of human PSC have progressed to a point that the generation of different lymphoid cell types for clinical implications is now on the horizon. In combination with CAR constructs, PSC‐derived CAR‐T cells and CAR‐NK cells have demonstrated promising antitumor functions in preclinical models. However, these differentiation systems do not completely reflect conventional lymphoid developmental pathways, suggesting there may be room for further improvements in protocol efficiency and fidelity.

## CONFLICT OF INTEREST

The authors have no conflicts of interest to declare.

## Data Availability

The data that support the findings of this study are available from the corresponding author upon reasonable request.
